# Relationships between psychosocial well-being and leisure time physical activity among 160.000 young Finnish men: a cross-sectional study during 2015–2021

**DOI:** 10.1186/s13690-023-01040-3

**Published:** 2023-02-17

**Authors:** Kaija Appelqvist-Schmidlechner, Risto Heikkinen, Tommi Vasankari, Toni Virtanen, Kai Pihlainen, Tuomas Honkanen, Heikki Kyröläinen, Jani P. Vaara

**Affiliations:** 1 Finnish Institute for Health and Welfare, Equality Unit, Helsinki, Finland; 2 Centre for Military Medicine, Helsinki, Finland; 3 Statistical Analysis Services, Analyysitoimisto Statisti Oy, Jyväskylä, Finland; 4 UKK Institute for Health Promotion Research, Tampere, Finland; 5 Faculty of Medicine and Health Technology, Tampere University, Tampere, Finland; 6 Finnish Defence Research Agency, Human Performance Division, Tuusula, Finland; 7 Defence Command, Training Division, Helsinki, Finland; 8 Department of Leadership and Military Pedagogy, National Defence University, Helsinki, Finland; 9 Faculty of Sport and Health Sciences, University of Jyväskylä, Jyväskylä, Finland

**Keywords:** Physical activity, Leisure time physical activity, Psychosocial well-being, Well-being, Self-esteem, Prosocial behaviour, Social relations, Men’s health

## Abstract

Evidence on the relationship between psychosocial well-being and physical activity (PA) is insufficient, especially in young adults between 18 and 29 years. Identifying protective factors for psychosocial well-being as well as factors that may promote PA behaviour in this specific age group is crucial.

The aim of the present study was to explore the association between self-reported leisure time physical activity (LTPA) and a number of measures of psychosocial well-being in a large sample of Finnish young men. The sample used for this study is based on registers of the Finnish Defence Forces and consist of questionnaire-based data collected from 159,776 young healthy men (18–29 years, mean age 19 years) who started their military service during the period from 2015 to 2021. Sum scores were calculated for self-esteem and prosocial behaviour. Physical activity, number of friends and the relationship with the parents were each measured with a single question. Unadjusted and adjusted (education, financial situation of the family, family structure) logistic regression models were calculated.

A multinominal logistic regression analysis showed that a higher level of LTPA was associated with a higher level of both prosocial behaviour (OR 6.12, 95% CI 5.88–6.36) and self-esteem (OR 4.41 95% CI 4.28–4.54). Further, LTPA had a positive relationship with good social relations, both with peers and parents. The odds ratio for participation in any LTPA weekly was higher (OR 2.74; CI 2.27–3.20) among those who had a close and trustworthy relationship with their parents compared to those men with more challenging relationships with their parents (OR 1.77; CI 1.46–2.14). An inactive lifestyle (PA less than once a week) seemed to be most common among men with no friends. About one third (31%) of men with no friends reported to engage in LTPA less than once a week, while only 10% of men with very many good friends reported to engage in similarly inactive LTPA behaviour.

LTPA seems to be positively associated with self-esteem, prosocial behaviour and good social relations among young adult men. Actions aimed at promoting LTPA may have a positive impact on psychosocial well-being among young men, or vice versa. The relationship between PA and psychosocial well-being is complex and interrelated.

## Introduction

Regular physical activity (PA) is beneficial for health. Besides maintaining and improving physical health [1, 2], it is well-established that PA is beneficial also for mental health [3–5]. However, less evidence exists on the social benefits of PA and on the relationship between PA and psychosocial well-being. Even though the definition of health according to the World Health Organisation [6] includes physical, mental and social health domains, social or psychosocial aspects and benefits of PA have been less frequently the focus of previous research.

The term psychosocial well-being has commonly been used as an umbrella term highlighting the close connection between psychological aspects of well-being and our wider social experience, including social and collective well-being [7], and it is commonly seen from two different perspectives: hedonic and eudaimonic well-being [8]. The hedonic perspective links well-being with happiness, affection and life satisfaction whereas the eudaimonic perspective highlights human potential that enable individuals to become involved and fulfilled [8].

According to previous research, PA and engagement in sport activities have the potential to improve both psychological and social well-being [9–12]. Previous reviews and meta-analyses generally support connections between PA and psychological and social outcomes, including social network and social relations, prosocial behaviour and self-esteem, both among young people and adults [11–17]. However, evidence on the relationship between leisure time physical activity (LTPA) and psychosocial aspects – such as self-esteem, prosocial behaviour and social relations – is inconclusive.

Self-esteem refers to a person’s evaluation of self-worth and it can be seen as an essential component of psychological well-being [18] associated with life satisfaction, positive affect, meaning in life and subjective vitality [19] as well as predicting success in different life domains such as social relations, work and health [20]. Self-esteem can be strengthened through personal traits, positive interpersonal relationship, collective social experiences [21] but also through PA [15, 17, 22]. This is explained by the skills development hypothesis [23] suggesting that an improved physical self-concept through participation in physical activities may lead to improved general self-esteem.

Besides benefits on self-esteem, PA is known to have various social benefits [10, 11, 24], for example, on social relations, social belonging and prosocial behaviour [13, 25–30]. These aspects are all also commonly related to each other [31]. Prosocial behaviour – defined as acts that are intended to benefit others [32] – has been indicated to have a positive association with PA, especially in men [25, 28]. The cross-sectional study by Wan et al. [25] found that higher levels of PA were associated with a higher likelihood of prosocial behaviour among high school students. Di Bartolomeo and Papa [28] evaluated the impact of short-term PA on prosocial behaviour in a randomized controlled trial and found that PA enhances trust and trustworthiness, and the effects did not seem to be temporary.

Furthermore, some previous studies have also found benefits of PA on social capital in terms of social relations and social integration [30, 33]. In a large sample of Finnish adults aged of 25–64 years [33], individuals who exercised at least twice a week experienced less cynical distrust and reported higher levels of a sense of coherence and a stronger feeling of social integration than their less frequently exercising counterparts. A study by Hoye et al. [30] found that participation in organized sport was associated with increased levels of social connectedness.

Social isolation and loneliness have been more frequently in the focus of interest. Vancamfort et al. [27] found an association between physical inactivity and higher odds for feeling lonely in a cross-sectional study with a large sample of adolescents aged between 12 and 15 years. Dos Santos et al. [34] found in their large national school-based health survey among Brazilian adolescents that more active adolescents were less likely to experience social isolation. The nature of the causal link still remains unclear. A review by Pels and Kleinert [13] pointed out that the beneficial effect of PA on loneliness depends on many factors and that loneliness itself can reduce the probability of being physically active. Haugen et al. [35] found that sport participation during adolescence is indirectly associated with lower level of loneliness through higher level of perceived social competence. Most of the studies in the field are based on samples with children and adolescents [27, 34–36] indicating a knowledge gap in this topic in adult populations.

Although some evidence already exists on the positive association between psychosocial well-being and PA [9–12], there is still insufficient knowledge on this relationship, especially in young adulthood. Given the previously exposed research gaps, the aim of the present study was to explore the association between self-reported LTPA and a number of measures of psychosocial well-being in a large sample of Finnish young men. The sample can be seen as population-based as it covers about 70% of entire male cohorts. Emerging adulthood – the transitional stage between late adolescence and young adulthood – is a crucial phase in the life span as multiple changes occur in this specific period of life. Identifying protective factors for psychosocial well-being as well as factors that may promote the PA behaviour in this specific age group is crucial. This knowledge can be used in developing interventions for promotion of health and well-being and prevention of psychosocial problems and ill-health in young adult males. In the present study, psychosocial well-being is investigated from the perspective of self-esteem, prosocial behaviour and social relations with peers and parents. It was hypothesized that individuals who engaged in more LTPA have higher level of self-esteem and prosocial behaviour, more friends and a better relationship with their parents.

## Material and methods

### Data collection

The data were collected among conscripts in the beginning of their military service in every fifteen unit across Finland between 2015 and 2021. In Finland, military or alternative civil service is obligatory for all Finnish men. Women can apply for military service on a voluntary basis. All male citizen are subjects to the conscription call-up starting from the beginning of the year in which they turn 18 years. At the call up, conscripts’ capability for service is assessed and the location and time of their service is determined. About 70–75% of men in each annual age cohort (about 23,000 men each year) start with their military service every year (Training Division of the Defence Command Finland, unpublished data). About 7% of each annual cohort choose civil service and 18–23% are exempted from the service already at the call up or through interruption of the service due to health reasons, mostly due to mental health or musculo-skeletal problems [37]. Out of the annual average of 23.000 men starting their military service, about 85% complete the service (Finnish Defence Forces: Annual Staff Report, 2021, unpublished).

The current study is a register-based study, where the data is based on questionnaires that all conscripts fill in as a part of their military service in the beginning of the service. The questionnaire has been developed originally and primarily for the purposes of developing the military training of the Finnish Defence Forces. The data collection was carried out within the first 2 weeks of the military service and the group of non-participants consisted most commonly of conscripts who had interrupted their service at the very beginning due to health or other reasons. The original data consisted of responses of 168,144 conscripts. Of these, 6.683 were female and 1.679 questionnaires contained no information on gender. Only questionnaires of male responses were included in the final sample to guarantee the representativeness of the sample. As carrying out the military service is voluntary for female conscripts, the sample of female respondents cannot be seen to represent the average young women of that age. After excluding 6 questionnaires without responses to any questions, the sample used for this study comprised of 159.776 study participants (mean age 19.7 years). This is 99% of all men who started with their military service during the period from 2015 to 2021.

### Measures

*Physical activity* was measured with a single question addressing LTPA during the last 2 months. LTPA was defined in the questionnaire as any leisure time physical activity with a minimum duration of 20 minutes. Response alternatives were the following: “Less than once a week”, “No vigorous but light/moderate LTPA at least once a week”, “Vigorous LTPA once a week”, “Vigorous LTPA twice a week”, “Vigorous LTPA 3 times a week” and “Vigorous LTPA at least 4 times a week”. This single question has been commonly used in various previous studies, for example, among Finnish reservists [38]. This single item question has been validated against fitness, observing that vigorous LTPA showed a fairly consistent dose-response relationship with cardiorespiratory and muscular fitness [39].

*Self-esteem* was measured with questions from the Rosenberg Self-Esteem Scale [40] including altogether 10 questions addressing global self-esteem: five items reflecting positive and five for negative feelings about oneself. As the main function of the survey was to serve the Finnish Defence Forces’ own purposes and information needs, instead of answering all items using the original 4-point Likert scale, a 5-point Likert scale from 1 (=strongly agree) to 5 (strongly disagree) was used as all other questions in the questionnaire used a 5-point scale. The aim was to keep the questionnaire as simply as possible for the respondents. The mean total score ranged between 1 to 5, with higher scores reflecting higher global self-esteem.

*Prosocial behaviour* was measured with the following six items reflecting social relations and social behaviour: 1) It is easy for me to make friends, 2) If I see someone to be in trouble, I go and help him/her, 3) It is comfortable for me to act in groups, 4) I commonly see following shared rules as important even if this would impose on me, 5) It is the duty of friends to help each other and 6) If a group has been given a task, everybody has to make their own contribution to the task. The responses were given with a 5-point Likert scale from 1 (=strongly agree) to 5 (strongly disagree). The mean total score ranged between 1 to 5, with higher scores reflecting higher level of prosocial behaviour. These questions have been developed and used for purposes of the Defence Forces and they have not been previously validated or used for purposes of academic research.

*Social relations* with parents and peers were measured with the following two questions: 1) “How is your relationship with your parents?” with response alternatives “good (close and trustful)”, “quite close”, “quite difficult” and “difficult” and 2) “How many friends did you have prior to military service?” with response alternatives “I have very many good friends”, “I have quite many good friends”, “I have a couple of or one good friend” and “I don’t have any good friends”. Both questions have not been previously validated or used for academic research.

The questionnaire also included questions about the education, family structure and financial situation of the family and this information was used as a background information in the study. No information about the age of each study participant was available, but it is known that conscripts in the present study were an average of 19,7 (range 18–29) years old, most commonly (95%) between 18 and 21 years (Training Division of the Defence Command Finland, unpublished data).

### Statistics

First, the Cronbach alpha values were calculated for the self-esteem and prosocial behaviour sum scores to measure the internal consistency of the measures. The Cronbach’s alpha of the total score was 0.87 for self-esteem and 0.71 for prosocial behaviour indicating acceptable internal consistency of the measures. Then, means, standard deviations (SD) and 95% confidence intervals (CI) for each PA intensity were calculated. Multinomial logistic regression models were used to test how prosocial behaviour and self-esteem predicted categorical PA variables with six levels. Odds ratios (OR) and 95% confidence intervals (CI) were calculated for self-esteem and prosocial behaviour according to the level of PA. Unadjusted analyses and adjusted analyses including the education (comprehensive school/vocational school/ high school and university) and financial situation of the family (low/moderate/high) were performed as education and the financial situation have been suggested to be associated with PA behaviour [41]. As an example, the interpretation of the result OR = 2 comparing engaging in vigorous PA once a week with engaging in vigorous PA less than once a week suggests that the odds for engaging in vigorous PA once a week (compared to less than once a week) is multiplied 2 times when prosocial behaviour increases by 1 unit. In terms of participation in LTPA according to different levels of social relation, logistic regression analyses were performed. The model for social relations was adjusted for the education of the participant (comprehensive school/vocational school/ high school and university) and financial situation of the family. The model for the relationship with parents was adjusted along with the education, financial situation of family (low/moderate/high) and family structure (both parents /other family structure).

The level of statistical significance was set to *p* < 0.05. Analyses were performed using R statistical software (R version 4.1.2).

## Results

The characteristics of the participants are presented in the Table 1. Most of the participants had a high school /university (44%) or vocational (45%) education and were living with both parents (79%). About one of third (34%) assessed the financial situation of their family as very good, 54% as moderate and 12% as poor. Three quarters (74%) of the participants were living in cities and 26% in the countryside. Almost one third (31%) of the participants reported having very many and 51% quite many good friends. Only 1% reported having no friends at all. Most of the men (81%) reported having a very and 17% quite good relationship with their parents. Only 2% assessed the relationship as difficult.

**Table 1:** Characteristics of the study sample among Finnish conscripts in 2015–2021

Variable	
Age	19.7 (mean)
	Distribution (%)
Highest education (*n* = 159.529)	
Comprehensive school or no education	8.7
Vocational school or training	46.5
High school / university	44.8
Family structure (*n* = 159.467)	
Father, mother and me (and siblings)	79.3
Other family structure	20.7
Financial situation of the family (*n* = 159.301)	
Quite low or low resources	12.2
Moderate resources	54.0
Very good or good resources	33.8
Domicile (*n* = 159.463)	
City with over 50.000 inhabitants	37.4
City with 20.000–50.000 inhabitants	21.6
City of under 20.000 inhabitants	14.9
Countryside	26.0
Social relationships (*n* = 159.411)	
I have very many good friends	31.2
I have quite many good friends	51.4
I have a couple of or one good friend	16.5
I do not have good friends	0.9
Relationship with parents (*n* = 159.383)	
Good (close and trustworthy)	81.1
Quite good	17.2
Quite difficult	1.3
Difficult	0.4
Leisure time physical activity (LTPA) (*n* = 159.515)	
Less than once a week	13.5
No vigorous but light/moderate PA at least once a week	22.6
Vigorous activity once a week	11.8
Vigorous activity twice a week	16.7
Vigorous activity 3 times a week	17.8
Vigorous activity at least 4 times a week	17.6

Of the study participants, 18% reported that they engaged in vigorous LTPA at least four times a week. More than one third (36%) of the men did not have any vigorous LTPA per week or were physically active less than once a week. About half of the men (48%) reported engaging in vigorous LTPA at most once a week.

Table 2 describes prosocial behaviour and self-esteem according to participation in LTPA. In the study sample, the mean for the prosocial behaviour sum score was 4.33 (SD 0.5, 95% CI 4.32–4.32) and for self-esteem 3.92 (SD 0.70, 95% CI 3.92–3.93). The higher the level of PA, the higher the score for both sum scores, indicating a higher level of self-esteem and prosocial behaviour (Table 2).

**Table 2:** Prosocial behaviour and self-esteem according to participation in leisure time physical activity (LTPA) among Finnish conscripts in 2015–2021

	Prosocial behaviour (***n*** = 158,585) Sum score mean (SD); 95% CI	Self-esteem (***n*** = 157,352) Sum score mean (SD); 95% CI
**LTPA**		
Less than once a week	4.07 (0.56); 4.06–4.07	3.49 (0.75); 3.48–3.50
No vigorous but light/moderate PA at least once a week	4.23 (0.49); 4.22–4.33	3.75 (0.69); 3.74–3.76
Vigorous activity once a week	4.30 (0.48); 4.29–4.30	3.89 (0.66); 3.88–3.90
Vigorous activity twice a week	4.37 (0.45); 4.36–4.37	4.00 (0.64); 4.00–4.01
Vigorous activity three times a week	4.43 (0.44); 4.43–4.44	4.11 (0.63); 4.10–4.12
Vigorous activity at least 4 times a week.	4.48 (0.45); 4.48–4.49	4.22 (0.62); 4.22–4.23

The odds ratios (OR) and 95% confidence intervals (CI) of LTPA with prosocial behaviour and self-esteem are presented in Table 3. LTPA was positively associated with prosocial behaviour and self-esteem. The multinominal logistic regression analysis in Table 3 shows that the likelihood of a higher level of LTPA increased with better self-esteem and prosocial behaviour in non-adjusted and fully adjusted models.

**Table 3:** The association of LTPA with prosocial behaviour and self-esteem (OR, 95% CI) among Finnish conscripts in 2015–2021

	Prosocial behaviour		Self-esteem	
	Model 1^a^ OR (95% CI)	Model 2^b^ OR (95% CI)	Model 1^a^ OR (95% CI)	Model 2^b^ OR (95% CI)
**LTPA**				
Less than one a week	1 (ref.)	1 (ref.)	1 (ref.)	1 (ref.)
No vigorous but light/moderate PA at least once a week	1.73 (1.68–1.78)	1.76 (1.71–1.81)	1.62 (1.59–1.65)	1.60 (1.57–1.63)
Vigorous activity once a week	2.27 (2.18–2.36)	2.33 (2.24–2.42)	2.17 (2.11–2.24)	2.08 (2.02–2.14)
Vigorous activity twice a week	3.13 (3.01–3.26)	3.26 (3.13–3.39)	2.81 (2.73–2.89)	2.62 (2.54–2.70)
Vigorous activity three times a week	4.44 (4.27–4.62)	4.63 (4.45–4.83)	3.67 (3.56–3.78)	3.32 (3.22–3.42)
Vigorous activity at least 4 times a week	5.89 (5.66–6.13)	6.12 (5.88–6.36)	5.11 (4.96–5.26)	4.41 (4.28–4.54)

*OR* Odds ratio, *95% CI* 95% confidence interval

^a^Unadjusted model

^b^Adjusted to include education (comprehensive school/vocational school/high school and university), and the financial situation (low/moderate/high) of the family

The Odds ratios (OR) and 95% confidence intervals (CI) for participation in leisure time physical activity (LTPA) according to friendship relations are presented in Table 4. LTPA had a positive relationship with good social relations, both with peers and parents. The results of the logistic regression analysis showed that the likelihood for weekly LTPA was higher with an increasing number of good friends. These associations were only modestly attenuated in the fully adjusted model.

**Table 4:** Odds ratios (OR) and 95% confidence intervals (CI) for participation in leisure time physical activity (LTPA) according to friendship relations (%) among Finnish conscripts in 2015–2021

	Model 1 OR (95% CI)	Model 2 OR (95% CI)
**Participation in any physical activity weekly**		
I have no friends	1 ref	1 ref
I have one of a couple of good friends	1.75 (1.55–1.96)	1.68 (1.48–1.91)
I have quite many good friends	2.86 (2.54–3.21)	2.62 (2.31–2.96)
I have very many good friends	4.12 (3.65–4.63)	3.68 (3.25–4.17)
**Participation in at least 3 times in vigorous physical activity per week**		
I have no friends	1 ref	1 ref
I have one or a couple of good friends	1.11 (0.98–1.27)	1.04 (0.91–1.20)
I have quite many good friends	1.75 (1.55–2.00)	1.59 (1.40–1.82)
I have very many good friends	3.05 (2.69–3.48)	2.77 (2.42–3.17)

Model 1 = unadjusted, Model 2 = adjusted with education (comprehensive school/vocational school/high school and university), and financial situation (low/moderate/high) of the family

**Fig. 1:**
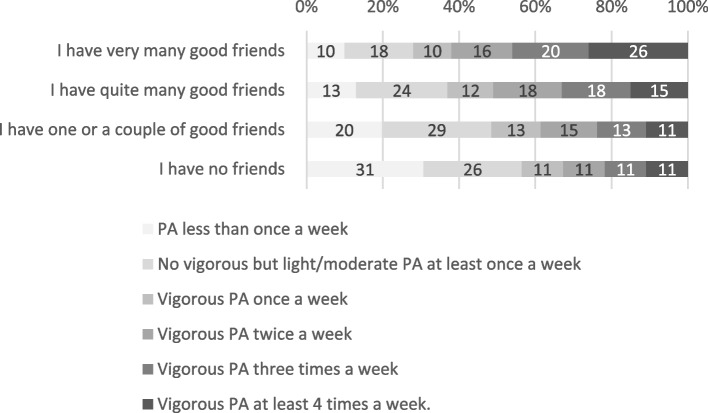
Level of leisure time physical activity (%) and reported number of friends (*n* = 159.411) among Finnish conscripts in 2015–2021

Interestingly, the likelihood of engaging in vigorous LTPA at least three times per week was higher in groups with quite or very many good friends, but not in the group with one or a couple of good friends (Table 4). These associations were only modestly attenuated in the fully adjusted model. An inactive lifestyle (LTPA less than once a week) seemed to be most common among men with no friends. About one third (31%) of men with no friends reported to engage in LTPA less than once a week, while only 10% of men with very many good friends reported to similarly inactive LTPA behaviour (Fig. 1.).

Table 5 presents the odds ratios (OR) and 95% confidence intervals (CI) for participation in LTPA according to relations with parents. The likelihood of engaging in any LTPA was higher within groups with a good or quite good relationship with their parents (Table 5). In terms of participating at least 3 times a week in vigorous LTPA, a statistically higher likelihood was observed only in the group reporting a close and trustworthy relationship with their parents, but only in the unadjusted model.

**Table 5:** Odds ratios (OR) and 95% confidence intervals (CI) for participation in leisure time physical activity (LTPA) according to relations with parents (%) among Finnish conscripts in 2015–2021

	Model 1 OR (95% CI)	Model 2 OR (95% CI)
**Participation in any LTPA weekly**		
Difficult relationship with parents	1 ref	1 ref
Quite difficult relationship with parents	1.32 (1.07–1.63)	1.06 (0.85–1.32)
Quite good relationship with parents	1.66 (1.37–1.99)	1.23 (1.09–1.49)
Close and trustworthy relationship with parents	2.74 (2.27–3.20)	1.77 (1.46–2.14)
**Participation at least 3 times in vigorous LTPA per week**		
Difficult relationship with parents	1 ref	1 ref
Quite difficult relationship with parents	1.01 (0.82–1.25)	0.83 (0.66–1.04)
Quite good relationship with parents	1.12 (0.93–1.36)	0.85 (0.70–1.05)
Close and trustworthy relationship with parents	1.74 (1.44–2.11)	1.19 (0.98–1.45)

Model 1 = unadjusted, Model 2 = adjusted with education, financial situation of family and family structure

## Discussion

Existing research on the social benefits of LTPA among young adults using large population-based data is limited and the evidence on this topic is inconclusive. The present study with a large Finnish sample of young men found that LTPA is positively associated with psychosocial well-being among young adult men. A positive relationship was found from the perspective of self-esteem, prosocial behaviour and social relationships. This association remained significant also after adjusting for socioeconomic background variables (education and financial situation of the family) that are suggested to be associated with physical activity so that a lower socioeconomic status is commonly associated with lower levels of physical activity [41].

The findings are in line with previously published studies [12, 22, 25, 28, 31]. In terms of self-esteem, support for the findings is provided by several previous research [15, 17, 22, 42]. The study by Resul Cekin [42] of young people in emerging adulthood, for example, showed that individuals who engage in regular PA are more likely to have higher self-esteem, optimism, and happiness than their physical inactive peers. One possible mechanism that may explain the positive relationship between PA and self-esteem is sport competence gained through PA. Perceived sport competence has been suggested to have an important mediating role in the relationship between PA and self-esteem [43]. The skills development model [23] hypothesizes that increased self-efficacy (i.e., beliefs in one’s personal capabilities) may lead to improved self-perception in various physical subdomains – for example relative to body attractiveness and sports competence – which in turn may increase global physical self-esteem [43]. A meta-analysis by Spence et al. [22] showed that engagement in PA indeed improves self-esteem depending on the change in physical fitness. However, the association can be also reversed: greater self-esteem may lead to more PA which is explained by the self-enhancement hypothesis [44]. Thus, self-esteem may influence PA and PA may influence self-esteem [31].

The present study showed that prosocial behaviour was also associated with LTPA. This finding also finds support in previous research [25, 28, 29, 45]. The direction of the causal link between PA and prosocial behaviour is – similarly to self-esteem – uncertain. Some evidence exists on the bidirectional relationship between PA and prosocial behaviour. Prosocial behaviour has been suggested to promote overall health [46] and to be a motivating factor for PA [47]. PA, vice versa, has been suggested to promote individual collective participation and interaction increasing empathy and trust between people and thereby enhancing individual prosocial behaviour [48]. Especially, team sport has been seen to support prosocial behaviours by creating a sense of belonging among team members and creating a social identity [12, 25].

Furthermore, the present study found a positive relationship between PA and social relations with peers and parents. Regular participation in PA was associated with a higher number of friends and a better relationship with one’s parents. Evidence on the association between the number of friends and PA is found mostly in studies using samples with children and adolescents indicating that a higher level of PA associates with a higher number of friends [36, 49]. For example, Jago et al. [49] investigated children’s PA in the transitional years between primary and secondary school and found that an extra friend was associated with almost four additional minutes of moderate to vigorous PA after school. The link was observed only for girls, but not for boys. Interestingly, it was observed in the present study that the likelihood of engaging in vigorous LTPA at least three times per week was higher in groups with quite or very many good friends, but not in the group with one or a couple of good friends, which may suggest that a physically active lifestyle is associated with a larger social network. Some evidence exists on the role of friends in engagement in PA. According to systematic reviews, friends’ PA levels have been seen to have a significant influence on an individual’s PA level both for young people [50], and adults [51].

Only 1 % of the sample in the present study reported having no friends at all. PA in this group of men was markedly less frequent compared to men with at least one friend. A systematic review by Pels and Kleinert [13] pointed out that PA can contribute to reduction in loneliness, but the beneficial effect of PA is dependent upon the quality of the relationships during PA. They also pointed out that loneliness itself may reduce the probability of being physically active. In the present study, unfortunately, no data was available on whether friends were involved in the weekly PA of study participants.

In terms of the relationship with the parents, some evidence exists on positive association between close child-parent relationships and PA [52] indicating a closer relationship to be associated with a higher level of self-reported PA behaviour. Some of the studies [53, 54] have investigated the association between perceived parental support and PA behaviour and found them to associate positive with each other. Perceived parental support has been suggested to play a crucial role in the PA behaviour of young people also in the longer term [53]. A longitudinal study by Doggui et al. [54] identified that parental support had long lasting associations with moderate-to-vigorous PA up to 5 years later. However, the findings of the present study rely only on one single question about the closeness of the child-parent relationship without any detailed information about perceived parental support. In the present study, the association between LTPA and the relationship with the parents was stronger from the perspective of engaging in any LTPA weekly. In terms of participating at least 3 times a week in vigorous LTPA, a statistically significant association was observed only for the group reporting the most closest and the most trustworthy relationship with their parents.

A notable finding was also that more than one third (36%) of the study participants reported not to engage in vigorous PA at all. This can be seen as a major public health concern considering the recommendations on PA by the World Health Organization [55]. A large-scale report by Väisänen et al. [56] has indicated a negative trend in cardiorespiratory fitness in the Swedish population and similar trend has also been observed in Finland [57]. Besides being a global public health concern, the lack of vigorous PA is also a special concern in the military context as, on the individual level, it may negatively affect the occupational performance, increase musculoskeletal injury risk and thus compromise overall training outcomes during service [58].

The mechanisms by which PA is associated with psychosocial well-being are not yet clear, but there are at least three potential hypotheses explaining the findings. First, participation in organized sports – even though the study focused more widely on LTPA – is known to provide a wide range of learning opportunities for social skills and prosocial behaviour, such as cooperation with others, respect for the rules and authority, self-control, dealing with disappointments and conflict resolution [16]. Second, participation in organized sport may have a positive effect on the sense of social inclusion also outside the sport, bonding with society in general and affecting positively prosocial behaviour and peer relations. The sense of belonging in a group or team practicing the same sport and the coaches as important role models are important factors that may influence the self-concept and psychosocial well-being positively [12, 59]. Third, as presented in the skills development hypothesis, PA may increase self-concept by increasing physical competence and positive body image which may positively influence the global self-esteem [43]. This, in turn, also may influence also prosocial behaviour and social relationships [31]. The relationship between PA, self-esteem, social behaviour and social relations can be seen as complex, dynamic and interrelated and should be investigated more closely in future studies.

### Strengths and limitations of the study

The present study uses a large population-based sample of Finnish young men which can be seen as a strength of the study. Our study population contains about 70% of Finnish 18–21 years old men from the period from 2015 to 2021 providing an exceptional study sample of young men. Young men in this age group can be regarded as a hard-to-reach group for studies using questionnaires as a research method [60, 61]. Thus, the results present valuable insights to the relationships between PA and the psychosocial well-being of young males in emerging adulthood. However, the study has also some limitations, which should be noted.

First, the main limitation is that the data used in this study has been collected primarily for purposes of the Finnish Defence Forces, not for academic research. Due to that, using validated scales in their original form for measuring psychosocial well-being has not been seen as that important, for example, in the case of the Rosenberg Self-esteem Scale. However, the focus of the present study was not on assessment of self-esteem of the study participants, but on relationship between PA and self-esteem. With regard to future use of these samples collected by the Finnish Military in academic research, it would be important to investigate the validity of the used questionnaire. Furthermore, the social relations with peers were measured with only one single question without any information about the intensity of closeness or context of the relationship, which would have provided deeper understanding of the role of friendships in the relationship with PA. Furthermore, LTPA was measured using one single self-reported question without any information about types of PA (i.e. individual, team sport etc). Self-reported LTPA data may differ from objective measured LTPA data and different types of LTPA may have different associations with psychosocial well-being as shown in previous studies [12, 25]. Furthermore, the data did not include information about the age of the study participants which meant that the age of the participants could not be used as a covariate in the regression models. However, due to the narrow age range of the respondents, mainly between 18 and 21 years, this can be seen only as a minor limitation. Second, the cross-sectional study design detects only associations between variables without information about causal relationships. More research is needed on the potential causal relationship between LTPA and psychosocial factors and mediating factors (such as the sociodemographic and family background) affecting this relationship. Third, even if the sample is population-based and large, a selection bias is to be taken into consideration. The sample consists of young men who have been assessed as healthy enough to carry out their military service. The data does not represent civil servants and men who have been exempted from the military or civil service due to health reasons. Men exempted from the military or civil service already at call ups and men who interrupt their service are known to have more psychosocial problems compared to those who carry out their military service [62]. Thus, men with poorer psychosocial and health status are underrepresented in the sample and this should be taken into consideration when generalizing the findings.

## Conclusions

LTPA is positively associated with self-esteem, prosocial behaviour and good social relations among young adult men. PA – especially when occurring with friends or in groups – provides possibilities for social interactions, sense of belonging and positive relationships with peers which may result also in increased self-esteem. Thus, actions aimed at promoting LTPA may also have a positive impact on psychosocial well-being. However, the relationship between PA and the psychosocial well-being among young adult males can be seen as complex and interrelated. More research with longitudinal and experimental study designs is needed to enhance the knowledge on this relationship.

## Data Availability

The datasets generated and analyzed during the current study are not publicly available but can be requested from the corresponding author with permission the Headquarters of the Finnish Defence Forces on reasonable request.

## Abbreviations

CI: Confidence interval
LTPA: Leisure time physical activity
OR: Odds ratio
PA: Physical activity
SD: Standard deviation
